# Mentalization in young patients undergoing opioid agonist treatment: Implications for clinical management

**DOI:** 10.1016/j.abrep.2023.100497

**Published:** 2023-05-18

**Authors:** Livia Pischiutta, Marco Garzitto, Giuliano Zamparutti, Enrico Moratti, Umberto Albert, Marco Colizzi, Matteo Balestrieri

**Affiliations:** a Unit of Psychiatry, Department of Medicine (DAME), University of Udine, 33100 Udine, Italy; b Drug Addiction Service, Department of Addiction, University Health Integrated Agency, 33100 Udine, Italy; c Department of Medicine, Surgery and Health Sciences, University of Trieste, 34149 Trieste, Italy; d Department of Psychosis Studies, Institute of Psychiatry, Psychology and Neuroscience, King's College London, London SE5 8AF, UK

**Keywords:** Substance use disorder, Opioid agonist treatment, Mentalization, Reflective functioning questionnaire

## Abstract

**Background:**

Mentalization is the ability to use internal mental states to manage and understand one’s own and others’ behavior. Inefficient mentalization has been associated to poor neuropsychological outcomes, including substance use disorder (SUD) and addiction. However, studies primarily investigating mentalization in SUD are lacking.

**Methods:**

Using the Reflective Functioning Questionnaire (RFQ), the Measurements in the Addictions for Triage and Evaluation, version 2.1 (MATE-IT-2.1), and the Mini International Neuropsychiatric Interview, 7th edition (MINI-7), an outpatient assessment investigated inefficient mentalization (i.e., ‘hypo-mentalization’ or ‘uncertainty’: concrete thinking with poor attribution of mental states; ‘hyper-mentalization’ or ‘certainty’: rigid and biased attribution of mental states) and socio-demographic and clinical characteristics, including SUD-related symptoms and any other psychiatric comorbidity, among opioid addiction (OA) patients in Opioid Agonist Treatment (OAT).

**Results:**

Thirty-seven consecutive OA patients in OAT (female, 45.9 %; age, M ± SD, 24.3 ± 3.55) were recruited. Patients’ mentalization differed from normative data, in terms of higher uncertainty and lower certainty scores. Also, higher uncertainty score was found among younger patients and in those with the most severe SUD in terms of craving and need for care. Finally, lower certainty score was found in those with a more severe substance abuse, previous contacts with pediatric mental-health services, and receiving a therapeutic community support.

**Conclusions:**

OA patients with inefficient mentalization present with a higher burden in terms of SUD severity, comorbidities, psychosocial disabilities, and service use, with important public health implications. Interventions targeting mentalization may have positive repercussions in preventing SUD, mitigating its severity, and containing its healthcare and social costs.

## Highlights

• Mentalization among opioid addiction (OA) patients differs from normative data.
• Younger patients and with the most severe disorder have higher uncertainty score.
• Patients with a more severe abuse have lower certainty score.
• Patients with previous pediatric mental-health contacts have lower certainty score.
• Patients receiving a therapeutic community support have lower certainty score.

## 1 Introduction

Substance Use Disorders (SUDs) have their onset before age 25 in 49 % of patients, and before age 18 in 15 % of them (Solmi et al., 2022), presenting with a high long-term clinical impact (Degenhardt and Hall, 2012). Among them, opioid addiction (OA) results in high rates of disability (Lusk and Stipp, 2018) and mortality (Hser et al., 2015). While Opioid Agonist Treatment (OAT; i.e., the controlled administration of opioid agonist drugs, with the main aim of reducing craving for the substance of abuse) has proven to be an effective treatment in controlling the disorder and reducing mortality risk (Hser et al., 2015, Santo et al., 2021), poor treatment adherence, post-treatment relapses, and risk behaviors remain unmet clinical needs (Krawczyk et al., 2020, Pearce et al., 2020), urging a better understanding of OA determinants to improve outcome.

Independently of biological susceptibilities (Deak and Johnson, 2021, Patriquin et al., 2015, Volkow et al., 2016), psychosocial risk factors have been suggested to contribute to the development of a SUD. A number of psychosocial factors have been explored, including, but not limited to, poor self-regulation, poor decision-making skills, and insecure attachment style (Gerra et al., 2021, Mishra et al., 2022, van Draanen and Aneshensel, 2022, Vungkhanching et al., 2004). Such evidence emphasizes the important role that interpersonal relationship and, more in general, social difficulties may play in sustaining SUD and OA.

Known for more than 30 years, the term mentalization refers to the ability to use internal mental states to manage and understand one’s own and others’ behavior (Fonagy and Luyten, 2009) at both the emotional and cognitive levels (Luyten et al., 2020). Generally, both excessively low use of mentalization (‘hypo-mentalization’, ‘uncertainty’ about mental states) and excessively high use (‘hyper-mentalization’, ‘certainty’ about mental states) are considered possibly dysfunctional. Mentalization is currently receiving much attention among studies because of the role that psychosocial determinants, such as interpersonal relationships, may have in altering its acquisition (Luyten et al., 2020), with implications for the development of mental disorders (Luyten et al., 2020). Indeed, among individuals exposed to dysfunctional social environments or traumatic events, especially in early childhood, the development of mentalization may be inhibited (Borelli et al., 2019). As a consequence, such individuals would present with a socially acquired (Sng et al., 2018) non-mentalistic interpretation of stimuli, based on representation of others’ behavior in terms of observable contents of experience (Gergely, 2003). The inability to process the cognitive and emotional components of social experience would lead to externalizing symptoms, such as impulsivity, inattention, interpersonal problems, and poor academic maturation (Fonagy and Luyten, 2018, Luyten and Fonagy, 2018, Perroud et al., 2017) as well as substance abuse (Luyten and Fonagy, 2018) and other addiction symptoms such as gambling (Cosenza et al., 2019), in a dysfunctional attempt to cope with stress and reduce arousal (Luyten et al., 2020).

To the best of our knowledge, studies investigating mentalization in SUDs, especially in patients with OA undergoing OAT, are lacking. The aim of this study was twofold: (i) to describe mentalizing abilities among OA patients undergoing OAT; (ii) to investigate the association between mentalization characteristics on one hand, and sociodemographic and clinical characteristics on the other, in this specific patient population. Mentalization impairments in patients receiving OAT were hypothesized, along with differential sociodemographic and clinical profiles as a function of mentalization abilities.

## 2 Materials and methods

### 2.1 Study design and sample

This study was conducted at the Drug Addiction Service of Friuli Centrale Health University Authority, Udine, Italy, an outpatient facility specifically devoted to the care of people experiencing SUDs. All young patients aged 18–30 years old, consecutively assessed for OA and treated with OAT over the period July to November 2021, were included into the study. Patients who were deemed clinically unstable (e.g., treatment stabilization not reached yet) as well as with a known or suspected acute substance intoxication were excluded.

The authors assert that the work described here has been carried out in accordance with The Code of Ethics of the World Medical Association (Declaration of Helsinki) for experiments involving humans as well as the Uniform Requirements for manuscripts submitted to biomedical journals.

### 2.2 Assessment

The assessment took place during the scheduled appointments. It included: (i) a general data collection form, with additional relevant information about the study participants being extracted from the clinical records; (ii) structured and semi-structured clinical interviews concerning the SUD-related symptoms and any other psychiatric symptoms/disorders; iii) a self-administered evaluation of mentalizing abilities. A brief description of adopted instruments is provided below.

*MATE-IT-2.1*. The Measurements in the Addictions for Triage and Evaluation, version 2.1 (MATE-IT-2.1) (Schippers et al., 2010), in its official Italian version (Schippers et al., 2013), was used. It is a structured interview for a multidimensional assessment of addiction problems. The MATE-2.1 provides quantitative measures of different clinical domains. This work focuses on five modules: (i) Substance use (S1, evaluating the main dependence issues experienced as well as the main substances of abuse; ii) Substance dependence and abuse (S4, measuring ‘Dependence’, ranging 0–7, ‘Abuse’, ranging 0–4, and ‘Severity of dependence/abuse’, ranging 0–9; iii) Activities and participation, care and support (S7, measuring ‘Total limitations’, ranging 0–76, ‘Basic limitations’, ranging 0–32, ‘Relationships limitations’, ranging 0–20, and ‘Care and support’, ranging 0–32; iv) Environmental factors influencing recovery (S8, measuring the external influences in terms of ‘Positive’, ranging 0–12, ‘Negative’, ranging 0–20, and ‘Need for care’, ranging 0–20), and (v) Craving (Q1, with a general score ranging 0–20).

*MINI-7*. The official Italian version of the Mini International Neuropsychiatric Interview, 7th edition (MINI-7), was used (Sheehan et al., 1998, Sheehan, 2016). It is a semi-structured interview assessing 17 psychiatric disorders according to DSM-5 diagnostic criteria.

*RFQ*. The Reflective Functioning Questionnaire (RFQ) (Fonagy et al., 2016), in its official Italian version, was used. RFQ studies conducted among Italian adults support its structure validity (Morandotti et al., 2018) and psychometric quality (Velotti et al., 2021). It consists of eight items scored on a 7-point Likert’s scale (from ‘Completely disagree’ to ‘Completely agree’), measuring two mentalizing dimensions: (i) ‘Uncertainty’ about mental states (RFQ-U), with high scores indicating hypo-mentalization (i.e., concrete thinking, with difficulties in understanding one’s own and others’ mental states; ii) ‘Certainty’ about mental states (RFQ-C), with high scores indicating hyper-mentalization (i.e., a rigid and biased attribution of mental states, that go far beyond available evidence). As an example, a person shows more uncertainty about others’ mental states if they agree with the statement: “People’s thoughts are a mystery to me”. On the other hand, a person stating “I always know what I feel” shows certainty with respect to one’s own mental states. The two scales are measured by assigning different weights to partially overlapping items, so that both scales are composed of six items and give a score ranging from 0 to 18.

### 2.3 Statistical analysis

Welch’s corrected t-tests were used for group comparisons with homoscedastic measures, and Mann-Whitney’s test in case of heteroscedasticity. Associations between continuous measures were analyzed with Pearson’s correlations (r), presented with their 95 % confidence intervals (CIs). All bivariate correlations were calculated on 37 observations. Fisher’s z tests were used to compare correlation coefficients. In correlation analysis, to control for multiple hypothesis testing, Benjamini-Hochberg’s procedure was adopted. Cronbach's αs for the RFQ scales were reported, with their 95 % CIs (calculated by Feldt's method).

In the main analysis, multiple linear regression models were then used on standardized measures to test for any association between variables, by correcting for the effects of potential confounders. Given the small sample size and the preliminary nature of this study, forward selection of predictors informed by Akaike's Information Criterion (AIC) was used. The initial pool of potential measures was chosen based on the statistically significant results of the preliminary analyses. For multiple linear regressions, a post-hoc power analysis was conducted, reporting in main-text only sub-optimal results (i.e., β > 0.20) in terms of achieved power for statistically significant results.

Model statistical significance (F or χ^2^), coefficients of determination (R^2^), and Cohen’s f as well as tolerances and predictor coefficients (βs, with 95 % CI) were reported. Statistical significance was set at α = 0.050, adopting two-tailed hypotheses. Analyses were conducted using R-4.2.1 software (R Development Core Team, 2022) and G*Power 3.1.9.7 (Faul et al., 2007).

## 3 Results

### 3.1 Recruitment process

Fifty-eight patients on OAT aged 18–30 years were considered for inclusion in the study. Of them, two were found not to be eligible, based on their medical records (i.e., one patient with cognitive disability, another patient with severe psychotic disorder). Further, thirteen patients were excluded because they were deemed clinically unstable by the referring clinicians. Finally, during the recruitment process, three patients moved to another location, one patient died, and another discontinued OAT. Informed consent was then offered to 38 patients. During the assessment period, a patient dropped out of the study before concluding all the evaluations, leaving a final sample size consisting of 37 participants.

### 3.2 Sociodemographic characteristics

Most patients were male (54.1 %), with a middle school qualification (51.4 %), living with their family of origin (45.9 %), and on a paid job (48.6 %). A substantial proportion of patients presented with medical comorbidities (35.1 %), family history of psychiatric (51.4 %) or substance use (43.2 %) problems, and current (45.9 %) or past (54.1 %) legal issues. All patients were tobacco smokers (Table 1).

**Table 1:** Socio-demographic characteristics.

Measures		Mean ± SD [range]/Frequency ( %)
Assessment:	Age (years)	24.26 ± 3.554 [18, 30]
Sex:	Female	17 (45.9 %)
Nationality:	Non-Italian	2 (5.4 %)
School qualification:	Middle school	19 (51.4 %)
	High school	16 (43.2 %)
	Degree	2 (5.4 %)
Number of failures at school:	Total	1.86 ± 1.337 [0, 6]
	Primary	0.03 ± 0.164 [0, 1]
	Middle	0.70 ± 1.051 [0, 4]
	High	1.14 ± 1.110 [0, 4]
Current housing:	Alone	2 (5.4 %)
	Family of origin	17 (45.9 %)
	New family	13 (35.1 %)
	Relatives or friends	1 (2.7 %)
	Other situations	4 (10.8 %)
Current job:	Paid job	17 (48.6 %)
	Student	5 (14.3 %)
	Housework	0 (0.0 %)
	Unemployed	13 (37.1 %)
Financial maintenance:	Family	14 (41.2 %)
	Autonomous (stable)	18 (52.9 %)
	Autonomous (unstable)	2 (5.9 %)
Medical comorbidity:	Presence	13 (35.1 %)
Family problems:	Psychiatric problem	19 (51.4 %)
	Substance abuse	16 (43.2 %)
Other mental-health Services:	Contact	14 (37.8 %)
	Start age (years)	14.82 ± 5.582 [5, 22]
Child/Adolescent psychiatry:	Contact	8 (22.2 %)
	Start age (years)	10.62 ± 4.307 [5, 15]
Legal/Judicial problems:	Current	17 (45.9 %)
	Past	20 (54.1 %)
	Prison	10 (27.0 %)
Tobacco:	Use	37 (100.0 %)
	Start age (years)	12.43 ± 2.489 [8, 23]

SD, Standard deviation.

### 3.3 Clinical characteristics

Most patients were receiving methadone as OAT (67.6 %; with a mean dose of 68.0 ± 47.28 mg and a mean duration of 3.4 ± 3.23 years), while the remaining participants were receiving suboxone (6.42 ± 5.931 mg/4 mg, 2.0 ± 2.49 years). Psychiatric comorbidity, as for DSM-5 criteria, was 86.5 %, with most patients presenting with antisocial personality (78.4 %), depressive disorder (64.9 %), and panic disorder (48.6 %). Suicidal risk (21.6 %) and history of traumatic life events (67.6 %) were found in a non-negligible proportion of patients (Table 2).

**Table 2:** Clinical characteristics.

Measures		Mean ± SD [range]/Frequency ( %)
At care at local Drug Addiction Service:	Entry age (years)	19.65 ± 3.039 [15, 27]
	Duration of caretaking (years)	4.12 ± 3.526 [0.06, 12.22]
	Current medical treatment	37 (100.0 %)
	Current psychological treatment	12 (32.4 %)
	Current group treatment	7 (18.9 %)
	Current community	6 (16.2 %)
	Past community	8 (21.6 %)
Opioid agonist therapy:	Methadone	25 (67.6 %)
	Suboxone	12 (32.4 %)
	High dosage	12 (32.4 %)
Methadone:	Dosage (mg)	67.96 ± 47.276 [15, 200]
	Start age (years)	20.76 ± 2.454 [17, 27]
	Duration (years)	3.36 ± 3.231 [0.40, 11.47]
Suboxone:	Dosage (mg/4 mg)	6.42 ± 5.931 [1, 16]
	Start age (years)	21.00 ± 4.045 [15, 27]
	Duration (years)	1.99 ± 2.492 [0.06, 9.20]
Urine, Opioids:	Positive < 1 month	9 (24.3 %)
	Positive 1–6 months	13 (35.1 %)
	Positive > 6 months	15 (40.5 %)
	Not-detectable	0 (0.0 %)
Urine, Cocaine:	Positive < 1 month	9 (24.3 %)
	Positive 1–6 months	9 (24.3 %)
	Positive > 6 months	15 (40.5 %)
	Not-detectable	4 (10.8 %)
Urine, Other substances of abuse:	Positive < 1 month	16 (43.2 %)
	Positive 1–6 months	6 (16.2 %)
	Positive > 6 months	13 (35.1 %)
	Not-detectable	2 (5.4 %)
Any DSM-5 diagnosis:	Lifetime	32 (86.5 %)
	Current	32 (86.5 %)
Comorbidity:	Number	2.76 ± 1.832 [0, 8]
	None	3 (8.1 %)
	Single	6 (16.2 %)
	More than one	28 (75.7 %)
DSM-5 diagnosis:	Antisocial personality	29 (78.4 %)
	Depressive disorder	24 (64.9 %)
	Panic disorder	18 (48.6 %)
	Generalized anxiety disorder	6 (16.2 %)
	Post-traumatic stress disorder	6 (16.2 %)
	Bulimia nervosa	5 (13.5 %)
	Bipolar disorder	2 (5.4 %)
	Obsessive-compulsive disorder	2 (5.4 %)
	Anorexia nervosa	1 (2.7 %)
	Social anxiety disorder	1 (2.7 %)
Current drug:	Any psychotropic	31 (86.1 %)
	Benzodiazepines	24 (64.9 %)
	Antipsychotics	13 (36.1 %)
	Antidepressants	10 (27.0 %)
	Stabilisers	2 (5.4 %)
	Other psychotropic	11 (29.7 %)
Suicidal risk:	Present	8 (21.6 %)
Traumatic life-events:	Reported	25 (67.6 %)

DSM-5, Diagnostic and Statistical Manual of mental disorders, version 5; MINI-7, Mini International Neuropsychiatric Interview, version 7, for DSM-5; SD, Standard deviation.

### 3.4 Mentalizing characteristics

**Fig. 1:**
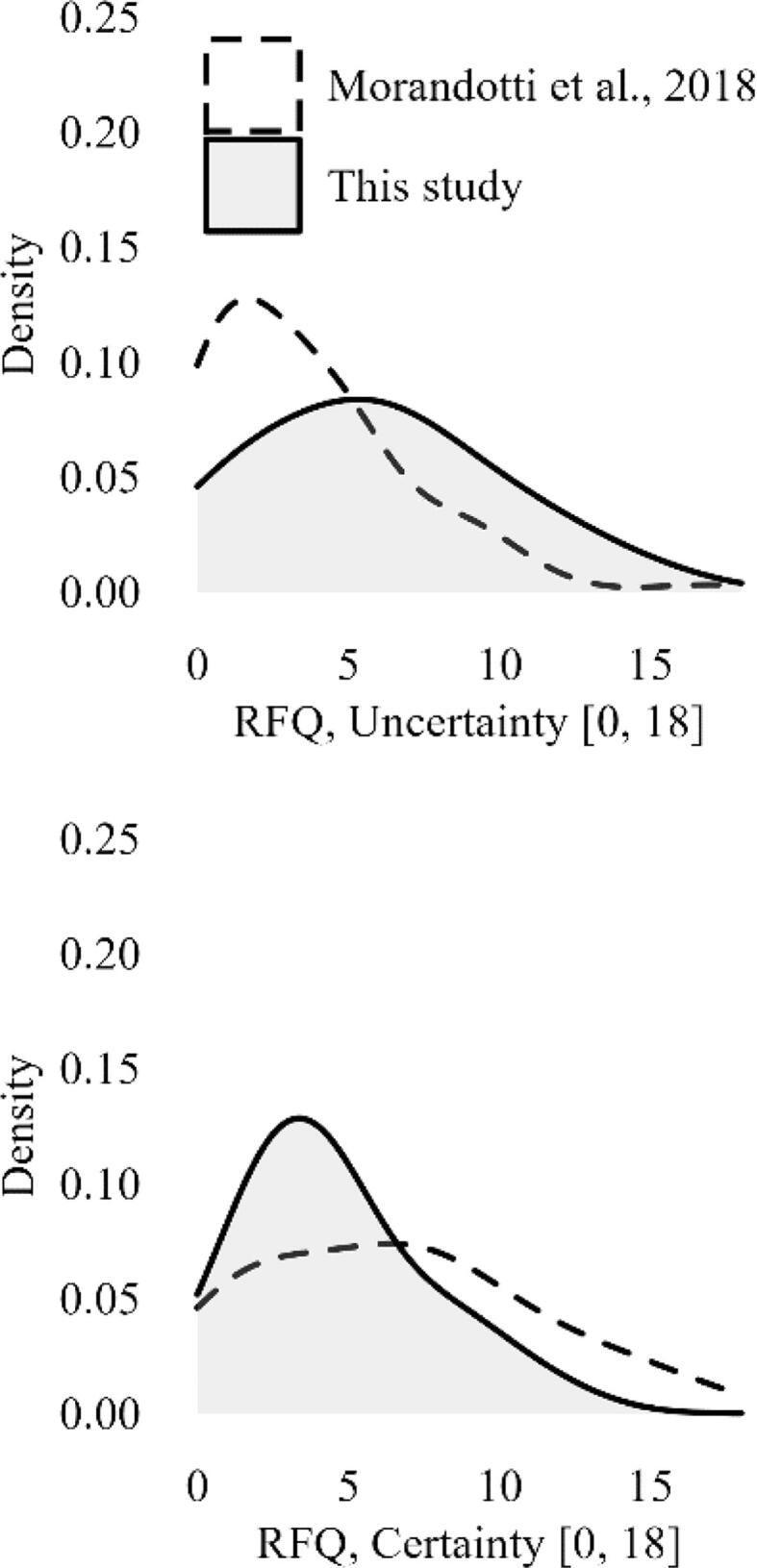
Distribution of mentalizing scores in this study and in normative data Normative data, age-matched nonclinical young adults (Morandotti et al., 2018); RFQ, Reflective Functioning Questionnaire.

Significant differences were found when comparing the mentalization skills measured in our sample (M ± SD standardized in z-score on normative scores: RFQ-U, +0.70 ± 1.193, t_49.9_ = −3.26, p = 0.002; RFQ-C, −0.43 ± 0.644, U = 1000.0, p = 0.033) with those referring to available normative data in non-clinical young adults (18–30 years; M ± SD: RFQ-U, 3.57 ± 3.335; RFQ-C, 6.50 ± 4.488) reported by (Morandotti et al., 2018). A graphical representation of the comparison between our sample and the normative sample is provided in Fig. 1. The two RFQ scales were negatively correlated in our sample (r = −0.496, 95 % CI: [−0.707, −0.205]), without being statistically different from the correlation obtained from normative data (-0.533 [−0.731, −0.252]; Fisher's z = +1.198, p = 0.231). The internal consistency for both RFQ scales was questionable: Cronbach’s α = 0.632, 95 % CI: [0.412, 0.789], for the RFQ-U scale, and 0.677 [0.485, 0.815] for the RFQ-C scale.

Univariate analyses, indicating associations between sociodemographic and clinical characteristics on one hand, and mentalizing characteristics on the other, are provided in the Supplementary results.

### 3.5 SUD characteristics

When assessing SUD-related symptoms, most patients reported difficulties in the opioid use domain (MATE-IT-2.1 S1: 28 participants, 75.7 %). Also, most patients presented with scores above cut-off and thus suggestive of dependence (S4.1: 20 participants, 54.1 %) and abuse (S4.2: 23, 62.2 %), with symptoms above severity cut-off presented by almost one third of the sample (S4.3: 12, 32.4 %). Less frequent were the above-threshold scores for craving (Q1: 4, 10.8 %), basic limitations (S7.2: 2, 5.4 %), and negative external influences (S8.2: 1, 2.7 %). Further details are provided in the Supplementary Table 1.

### 3.6 Association between SUD and mentalizing characteristics

Univariate analyses, indicating associations between SUD and mentalizing characteristics, are provided in the Supplementary results and Supplementary Table 1. Possible predictors for RFQ-U scale were as follows: age, information on DSM-5 diagnosis (number of comorbidities, presence of Generalized Anxiety Disorder, presence of Post-Traumatic Stress Disorder), reported traumatic life-events, and scores from MATE-IT-2.1 (S4.1, S4.2, S4.3, S7.1, S7.3, S8.2, S8.3, and Q1). Instead, for RFQ-C scale they were as follows: previous contacts with child/adolescent mental health services, having had failures at school, caretaking at local Drug Addiction Service (duration of service use, current therapeutic community), and scores from MATE-IT-2.1 (S4.2, S4.3, S7.1, S7.2, S7.3, S7.4, and S8.3).

AIC-based stepwise forward selection produced a statically significant overall final model for the RFQ-U scale (F_4,33_ = 8.96, p < 0.001; R2 = 0.449; Cohen’s f = 0.903), with AIC decreasing from + 107.99 to +91.94). MATE-IT-2.1 predictors added to the model were craving (Q1, β = +0.388, 95 % CI: [+0.109, +0.666], p = 0.008; tolerance: 0.888) and need for care (S8.3, +0.297 [+0.009, +0.585], p = 0.044; 0.833; with a low estimated power: 57.8 %). Generalized Anxiety Disorder (GAD) diagnosis was also included in the model as a predictor, although without statistical significance (+0.599 [−0.173, +1.000], p = 0.124; 0.829; Table 3).

**Table 3:** Multiple linear regression models for RFQ scales.

Predictor	β	[95 % ci]	p
**Model for *Uncertainty scale* (RFQ)**			
Craving score (MATE-IT-2.1, Q1)	+0.388	[+0.109, +0.666]	0.008*
Need for care score (MATE-IT-2.1, 8.3)	+0.297	[+0.009, +0.585]	0.044*
Possible diagnosis of GAD	+0.599	[−0.173, +1.000]	0.124
**Model for *Certainty scale* (RFQ)**			
Abuse score (MATE-IT-2.1, S4.2)	−0.317	[−0.581, −0.053]	0.020*
Limitations - Total score (MATE-IT-2.1, S7.1)	−0.376	[−0.753, +0.001]	0.051
Need for care score (MATE-IT-2.1, S8.3)	−0.007	[−0.414, +0.400]	0.971
Previous child/adolescent mental-health services	−0.826	[−1.000, −0.270]	0.005*
Current therapeutic community care	−0.765	[−1.000, −0.053]	0.036*
School failures	+0.585	[−0.068, +1.000]	0.077

Akaike’s Information Criterion (AIC)- based forward selection of predictors; ci, Confidence interval; MATE-IT-2.1, Measurements in the Addictions for Triage and Evaluation, Italian version 2.1; RFQ, Reflective Functioning Questionnaire; *, Statistically significant with p < 0.050.

The overall final model for the RFQ-C scale was also statistically significant (F_7,30_ = 7.31, p < 0.001; R2 = 0.594; Cohen’s f = 1.209), with AIC decreasing from + 107.99 to + 86.66). MATE-IT-2.1 predictor added to the model was substance abuse (S4.2, −0.317 [−0.581, −0.053], p = 0.020; tolerance: 0.810; with a low estimated power: 74.9 %). Total limitations (S7.1, −0.376 [−0.753, +0.001], p = 0.051; 0.397) and need for care (S8.3, −0.007 [−0.414, +0.400], p = 0.971; 0.341) were also included in the model as predictors, although without statistical significance. Further predictors for the RFQ-C scale were past child/adolescent mental-health service care (-0.826 [−1.000, −0.270], p = 0.005; 0.966), current therapeutic community care (-0.765 [−1.000, −0.053], p = 0.036; 0.798; with a low estimated power: 65.5 %), and history of school failures (+0.585 [−0.068, +1.000], p = 0.077; 0.948), with the latter narrowly missing statistical significance (Table 3).

## 4 Discussion

Findings from this study indicate difficulties in mentalization, in terms of higher uncertainty and lower certainty, among patients receiving OAT for their opioid addiction, compared to the profile of normative data from an age-matched non-clinical sample. Also, higher uncertainty was found among younger patients and in those with the most severe substance use disorder (SUD) in terms of craving and need for care. Finally, lower certainty was found in those with a more severe substance abuse, previous contacts with pediatric mental-health services, and receiving a therapeutic community support.

When exploring characteristics associated with altered mentalization, both direct (e.g., opioid abuse, need for care) and indirect (e.g., previous pediatric mental-health contacts, school failures) psychosocial features among SUD patients were found accompanying altered mentalizing patterns, in line with theoretical models of aberrant mentalization development (Fonagy and Luyten, 2009, Luyten et al., 2020). Specifically, in the context of such psychosocial difficulties, OA patients presented higher scores on the uncertainty scale, that reflects hypo-mentalization, an interpretation of social experiences mainly based on a non-mentalistic observation of others’ behaviors (Gergely, 2003). Further, results from this study confirmed and extended previous evidence of an association between mentalizing difficulties and mental health symptoms more in general, believed to result from an inefficient social processing of cognitive and emotional stimuli (Fonagy and Luyten, 2018, Luyten and Fonagy, 2018). In fact, greater hypo-mentalization was found among SUD patients also suffering of GAD, possibly as a dysfunctional response to stress and hyperarousal states (Borelli et al., 2019, Luyten et al., 2020).

Certainty, whose extreme scores would indicate a dysfunctional hyper-mentalization, i.e., an interpretation of others’ behaviors without evidence supporting that presumption, was lower in patients with more severe SUD and related psychosocial disabilities. When assessing the multifaceted components of mentalization, research evidence indicates that uncertainty scores show a positive correlation with alexithymia (i.e., difficulties in identifying, understanding, and describing emotions), and a negative correlation with empathy (i.e., understanding the mental states of others while resonating with them) and mindfulness (i.e., awareness of what one experiences). Instead, certainty scores would show the opposite pattern, correlating negatively with alexithymia and positively with empathy and mindfulness (Badoud et al., 2015). In line with these findings, our results would suggest poor mentalizing abilities, in terms of reduced empathy and mindfulness as well as increased alexithymia, among OA patients presenting with greater abuse, service use, and psychosocial difficulties.

Results from this study must be seen considering its strengths and limitations. To the best of our knowledge, empirical evidence about mentalizing abilities in SUD is lacking, making the present findings valuable for clinicians and researchers working in the field. Also, a clinically well-defined sample was obtained, to limit the implications of “spurious comorbidity”, that is the higher co-occurrence of disorders in clinically ascertained samples than in population-based samples, possibly due to patients presenting with multiple conditions being more likely to seek medical care and receive a diagnostic evaluation. However, the limited sample size, obtained in a single recruitment hub, suggests caution in drawing conclusions about mentalizing abilities in SUD, requiring replication of the findings in larger multisite studies. Still referring to the small sample-size, we emphasized that the power calculated a posteriori for some results was sub-optimal (i.e., for some coefficients of the multiple regression models). Also, participants included in the study were patients in frequent contact with clinicians for their therapeutic needs, with regular medical check-ups, and adherence to health-care pathways. While offering advantages in terms of recruitment feasibility and sample homogeneity, this may have similarly affected the generalizability of the results to the wider population of individuals with OA. Also, data collection was carried out during the pandemic restrictions for COVID-19. Although restrictions were reduced during the assessment period, we have no way of assessing whether the reported data may have been influenced by the atypical contextual situation. Finally, the RFQ that was used to assess mentalization characteristics in our sample, has been criticized. Also, in our observation the scales of the RFQ showed poor internal consistency. It has been suggested that, despite reducing administration time, administrator training, and burden on participants (Fonagy et al., 2016), its psychometric reliability may be lower when compared to the Reflective Functioning Interview, the gold standard measure for the assessment of mentalization (Anis et al., 2020, Morandotti et al., 2018). Arguably, at the psychometric level, the RFQ seems to be better suited at capturing a single dimension of dysfunctional mentalization style (Müller et al., 2022). Nevertheless, in our study we preferred to refer to the available Italian norms (i.e., organized in the two scales originally proposed), to allow a preliminary assessment of this poorly investigated area and a comparison with other observations available in the literature. However, these observations need further and deeper investigation, using more reliable assessment instruments, probably based on clinical interviews. In addition to a more reliable assessment of the characteristics of mentalization in patients with OA, future investigations may also clarify any associations with other psychiatric problems in comorbidity. For this, larger sample sizes will be needed to assess interactive effects between different disorders.

In conclusion, this study provides preliminary observations in an under-explored field, that is the evaluation of mentalizing abilities in SUD and its clinical course. Mentalization patterns among OA patients undergoing OAT were found to differ from what expected based on normative data. Also, patients with greater mentalizing dysfunction present with a higher burden in terms of SUD severity, comorbidities, psychosocial disabilities, and service use. Thus, findings may have important public health implications, as they suggest that interventions targeting mentalization may have positive repercussions in preventing SUD, mitigating its severity, and containing its healthcare and social costs.

## Contributors

Substantial contributions to the conception or design of the work and/or the acquisition, analysis, or interpretation of data for the work: all authors. Drafting of the manuscript and/or revising it critically for important intellectual content: all authors. Final approval of the version to be published: all authors. Agreement to be accountable for all aspects of the work in ensuring that questions related to the accuracy or integrity of any part of the work are appropriately investigated and resolved: all authors.

## Declaration of Competing Interest

The authors declare the following financial interests/personal relationships which may be considered as potential competing interests: M.C. has been a consultant/advisor to GW Pharma Limited, GW Pharma Italy SRL, and F. Hoffmann-La Roche Limited, outside of this work. All the other authors declare no conflict of interest.

## Data Availability

Data will be made available on request.
