# Supplementary material for: Mentalization in young patients undergoing opioid agonist treatment: Implications for clinical management
Source: Addict Behav Rep. 2023 May 18;17:100497. doi: 10.1016/j.abrep.2023.100497 (PMC10212784; doi:10.1016/j.abrep.2023.100497)
Supplement: Supplementary data 1 [file mmc1.docx]

**Supplementary Results**

*Association between sociodemographic, clinical, and mentalizing characteristics*

Considering patients’ sociodemographic characteristics, the RFQ-U scale was found to be associated with younger age (-0.381 [-0.627, -0.065]), while the RFQ-C scale was not (+0.265 [-0.065, +0.542]). Participants who had been followed by child/adolescent mental-health services showed lower scores on the RFQ-C scale (nine participants; 3.00±1.803 vs 5.11±3.010; t_23.2_=+2.55, p=0.018), but not on the RFQ-U scale (t_10.8_=-0.81, p=0.438). Also, there was a negative association between age of admission to other services and the RFQ-C scale (-0.839 [-0.957, -0.481]), but not with the RFQ-U scale (+0.478 [-0.171, +0.838]). Further, participants reporting school failures had lower scores on the RFQ-C scale (31 participants; 3.00±0.894 vs 4.90±3.048; t_28.6_=-2.89, p=0.007) but not on the RFQ-U scale (t_6.5_=+0.77, p=0.469).

Differences were also observed for clinical variables, both in terms of ongoing treatments and comorbidities. Patients placed in a therapeutic community (six patients; 16.2%) showed lower scores on the RFQ-C scale (2.33±1.366 vs 5.03±2.915; t_15.7_=+3.53, p=0.003), with no differences on the RFQ-U scale (t_6.5_=-0.87, p=0.416). Also, the duration of service use positively correlated with the RFQ-C scale (+0.358 [+0.038, +0.611]), but not with the RFQ-U scale (-0.303 [-0.571, +0.023]).

Further, a higher score on the RFQ-U scale was measured for patients suffering of Generalized Anxiety Disorder (GAD; six patients; 9.83±2.714 vs 5.13±3.748; t_9.2_=-3.63, p=0.005), Post-Traumatic Stress Disorder (PTSD; six participants; 9.50±3.391 vs 5.19±3.736; t_7.6_=-2.80, p=0.025), and in those reporting traumatic life events (25 participants; 6.68±4.337 vs 4.25±2.527; t_33.4_=-2.14, p=0.039). No significant differences were observed when considering RFQ-C scale scores (GAD: t_8.2_=+0.99, p=0.353; PTSD: t_20.2_=+1.49, p=0.152; traumatic life events: t_19.5_=+0.44, p=0.662). The RFQ-U scale was also associated with a higher number of comorbidities (+0.442 [+0.138, +0.670]), in contrast to the RFQ-C scale (-0.203 [-0.494, +0.130]).

*Association between SUD and mentalizing characteristics*

When correlating MATE-IT-2.1 and RFQ scores, after Benjamini-Hochberg’s correction for multiple comparisons, the duration of substance use was not associated with mentalizing abilities. Instead, the RFQ-U scale score was positively associated with main symptomatology (S4.1/2/3), limitations, particularly in relationships (S7.1/3), negative external influences and care needs (S8.2/3), and craving (Q1), with correlation coefficients in the low-to-moderate range (+0.443 ≤r ≤+0.531). Further, the RFQ-C scale score showed low-to moderate sized negative correlations (-0.511 ≤r ≤-0.382) with abuse and severity scores (S4.2/3), all scores in the activity and participation domain (S7.1/2/3/4) and need for care (S8.3; Supplementary Table 1).

**Supplementary Table 1.** Association between SUD and mentalizing characteristics

|  |  | **Mean ±SD [range]** | **r [95% ci]** | |
| --- | --- | --- | --- | --- |
| **Measure (MATE-IT-2.1)** |  | **/ Frequency (%)** | **RFQ-U** | **RFQ-C** |
| *Primary-problem substance*: | *Opioids* | 28 (75.7%) | - | - |
| (**S1**) | *Cocaine* | 7 (18.9%) | - | - |
|  | *Sedative* | 1 (2.7%) | - | - |
|  | *Alcohol* | 1 (2.7%) | - | - |
| *Years of regular use*: | *Opioids* | 4.43 ±2.395 [0.5, 10] | +0.060 [-0.269, +0.377] | +0.237 [-0.095, +0.521] |
| (**S1**) | *Cocaine* | 1.80 ±2.500 [0.0, 8] | +0.141 [-0.192, +0.445] | +0.013 [-0.312, +0.336] |
|  | *Stimulants* | 1.30 ±2.089 [0.0, 8] | -0.047 [-0.365, +0.281] | -0.094 [-0.406, +0.237] |
|  | *Ecstasy* | 0.96 ±1.976 [0.0, 8] | -0.014 [-0.336, +0.312] | +0.013 [-0.313, +0.335] |
|  | *Sedative* | 0.69 ±1.871 [0.0, 8] | +0.048 [-0.281, +0.366] | -0.258 [-0.537, +0.072] |
|  | *Cannabis* | 5.64 ±4.096 [0.0, 16] | +0.151 [-0.181, +0.453] | -0.040 [-0.360, +0.287] |
|  | *Gambling* | 0.15 ±0.676 [0.0, 4] | -0.015 [-0.337, +0.311] | -0.004 [-0.327, +0.321] |
|  | *Alcohol* | 1.24 ±2.209 [0.0, 10] | +0.074 [-0.256, +0.389] | +0.038 [-0.289, +0.358] |
|  | *Nicotine* | 9.58 ±3.457 [4.0, 16] | -0.094 [-0.406, +0.237] | +0.158 [-0.175, +0.458] |
|  | *Other* | 1.02 ±2.081 [0.0, 8] | +0.052 [-0.276, +0.370] | -0.127 [-0.433, +0.205] |
| *Substance dependence and abuse*: | **.1**, *Dependence score* | 3.32 ±3.010 [0, 7] | +0.493 [+0.201, +0.704]* | -0.371 [-0.620, -0.053] |
| (**S4**) | cut-off | 20 (54.1%) | - | - |
|  | **.2**, *Abuse score* | 1.57 ±1.385 [0, 4] | +0.480 [+0.185, +0.696]* | -0.475 [-0.692, -0.178]* |
|  | cut-off | 23 (62.2%) | - | - |
|  | **.3**, *Severity of dependence/abuse score* | 4.24 ±3.677 [0, 9] | +0.503 [+0.214, +0.711]* | -0.440 [-0.669, -0.135]* |
|  | cut-off | 12 (32.4%) | - | - |
| *Activities and participation; care and support*: | **.1**, *Limitations - Total score* | 13.70 ±8.313 [0, 31] | +0.483 [+0.188, +0.698]* | -0.458 [-0.681, -0.158]* |
| (**S7**) | **.2**, *Limitations - Basic score* | 3.81 ±3.471 [0, 12] | +0.381 [+0.065, +0.627] | -0.398 [-0.640, -0.085]* |
| Consistent with the ICF | cut-off | 2 (5.4%) | - | - |
|  | **.3**, *Limitations - Relationships score* | 4.32 ±2.719 [0, 10] | +0.450 [+0.148, +0.676]* | -0.403 [-0.643, -0.091]* |
|  | **.4**, *Care and support score* | 3.46 ±4.154 [0, 17] | +0.183 [-0.150, +0.479] | -0.382 [-0.628, -0.066]* |
| *Environmental factors influencing recovery*: | **.1**, *Positive external influences score* | 3.41 ±2.114 [0, 8] | -0.100 [-0.411, +0.231] | +0.055 [-0.274, +0.372] |
| (**S8**) | **.2**, *Negative external influences score* | 3.86 ±3.172 [0, 14] | +0.443 [+0.139, +0.671]* | -0.333 [-0.593, -0.010] |
| Consistent with the ICF | cut-off | 1 (2.7%) | - | - |
|  | **.3**, *Need for care score* | 8.38 ±3.577 [3, 16] | +0.485 [+0.191, +0.699]* | -0.511 [-0.717, -0.224]* |
| *Craving*: | *Craving score* | 3.84 ±5.565 [0, 18] | +0.531 [+0.250, +0.730]* | -0.339 [-0.597, -0.017] |
| (**Q1**) | cut-off | 4 (10.8%) | - | - |
| **RFQ**: | **U**, Uncertainty scale | 5.89 ±3.978 [0, 15] | - | -0.533 [-0.731, -0.252]* |
|  | **C**, Certainty scale | 3.08 ±1.754 [0, 8] | -0.533 [-0.731, -0.252]* | - |

| r, Bivariate Pearson’s correlation; ci, Confidence interval; ICF, International Classification of Functioning, Disability, and Health; MATE-IT-2.1, Measurements in the Addictions for Triage and Evaluation, Italian version 2.1; RFQ, Reflective Functioning Questionnaire; U, Uncertainty; C, Certainty; SD, Standard deviation. *: Statistically significant with p<0.050 after Benjamini-Hochberg's correction. |
| --- |
